# Open-source force analyzer with broad sensing range based on an optical pickup unit

**DOI:** 10.1016/j.ohx.2022.e00308

**Published:** 2022-04-20

**Authors:** Tien-Jen Chang, Line Hagner Nielsen, Anja Boisen, En-Te Hwu

**Affiliations:** The Danish National Research Foundation and Villum Foundation's Center for Intelligent Drug Delivery and Sensing Using Microcontainers and Nanomechanics (IDUN), Department of Health Technology, Technical University of Denmark, 2800 Kgs. Lyngby, Denmark

**Keywords:** Optical pick-up unit, 3D printing, Mucoadhesion, Cantilever, Force transducer, Microdevices

## Abstract

In the pharmaceutical field, oral drug delivery devices continue to shrink down to the micrometer scale, driving a trending demand to investigate *ex vivo* mucoadhesive force down to the micro-Newton scale. However, owing to the limitation of measuring sensitivity, conventional methods (e.g., a texture analyzer) lack reliability while measuring forces in this range. Herein, we report on an open-source force analyzer that utilizes an optical-pickup-unit (from a DVD player) to detect cantilever-based force transducers and thereby, achieves a wide force-sensing range from 1.1 N to 0.99 nN. The cantilever force transducers can easily be adjusted to fit different force ranges by adjusting the steel shim, magnets, and 3D printed components. To validate the analyzer, we conducted a preliminary study to investigate the effect of time and humidity of mucoadhesion of porcine intestinal tissues. Besides, we measured the mucoadhesive force of a single oral drug delivery microdevice with an average force of 93.7 µN on the top sides of the device. This analyzer offers the possibility of measuring e.g. mucoadhesion of individual microdevices in the micro-Newton range. Hence, the analyzer can assist in the development of miniaturized oral drug delivery devices but has a much wider field of potential force sensing applications.

## Graphical abstract

**Figure:**
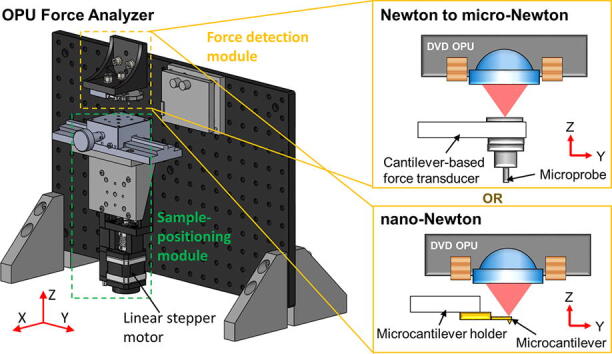

Specifications table.

**Table:** 

Hardware name	OPU force analyzer
Subject area	• Medical (e.g., Pharmaceutical Science) • Engineering and Material Science • Educational Tools and Open Source Alternatives to Existing Infrastructure • General
Hardware type	• Measuring physical properties and in-lab sensors • Mechanical engineering and materials science
Open-source License	*CC BY 4.0*
Cost of Hardware	*3000 – 3500 EUR*
Source File Repository	https://doi.org/10.17632/cnkd95kp65.1

## Hardware in context

In the past decades, different micrometer-scale devices have been proposed to improve the efficiency of oral drug delivery. These devices include microcontainers (MCs) for achieving a higher oral bioavailability of e.g. poorly water-soluble drugs [1], [2] and shape-changing microdevices to increase the retention time in the gastrointestinal tract (GI tract) as well as a delay in drug release [3]. The trend of shrinking the device size from the millimeter to micrometer scale leads to an unmet need to evaluate the mucoadhesive properties *in vitro* and *ex vivo* of microdevices.

The ability for oral drug delivery devices to be mucoadhesive in the GI tract is crucial as it influences the retention time of the devices as well as the absorption and oral bioavailability of the drugs [4], [5], [6]. In general, to characterize mucoadhesion, a flow-through [7] and a tensile strength method [8] have been used to investigate the interaction between a mucus layer and devices. The flow-through method simulates a transmit process by flushing the devices on intestinal tissue. Observing the flowing characteristics among different devices provides a comparative result of the mucoadhesion. However, the flow-through method cannot quantify the mucoadhesive force. The tensile strength method directly measures the mucoadhesion force of a single device, illustrating the mucoadhesion by a force-to-displacement curve [9]. The most widely used instrument for detecting mucoadhesion force is a texture analyzer, which provides force measurements in the range from Newton to milli-Newton [4], [9], [10]. However, the mucoadhesive forces of most microdevices are in the micro-Newton range. As a result, the texture analyzer cannot provide reliable measurements for microdevices. The texture analyzer can switch to a more sensitive load cell to achieve a higher force measurement resolution. However, the mechanical framework design of the texture analyzer and positioning mechanism introduces internal vibrations that interfere with force sensing in the micro-Newton range [10].

Atomic force microscopy (AFM) can achieve high force measurement sensitivity in the nano-Newton range. Thus, an AFM was modified to detect the mucoadhesion of nanoparticles [11]. Nevertheless, limited by the fragile microscale cantilever, AFM can only carry a maximum sample size in the range of tens of micrometers. Current force measurement methods cannot provide reliable characterizations of mucoadhesion force at the micro-Newton scale for micrometer-sized particles and devices. Other commercial microscale tensile force instruments may potentially be repurposed for this measurement task. However, most of these costly instruments are not available as open sources, making it challenging to modify them according to customized applications [12].

An optical-pickup-unit (OPU) is an electronic component for recording and retrieving digital data on optical disks such as CDs, DVDs, HD DVDs, and Blu-rays [13]. An OPU integrates a laser source, sensors, actuators, and delicate optics in a compact size, similar to a matchbox. Benefited from mass production for the optical data storage market, an OPU unit is priced at around 5 US$. Furthermore, the low-cost OPU can achieve nano-meter displacement sensitivity from 0.1 Hz to MHz signal bandwidth. OPUs have been successfully utilized for various applications, such as AFMs [14], [15], [16], stereolithography 3D printing [17], photolithography [18], and biosensing [19].

**Fig. 1:**
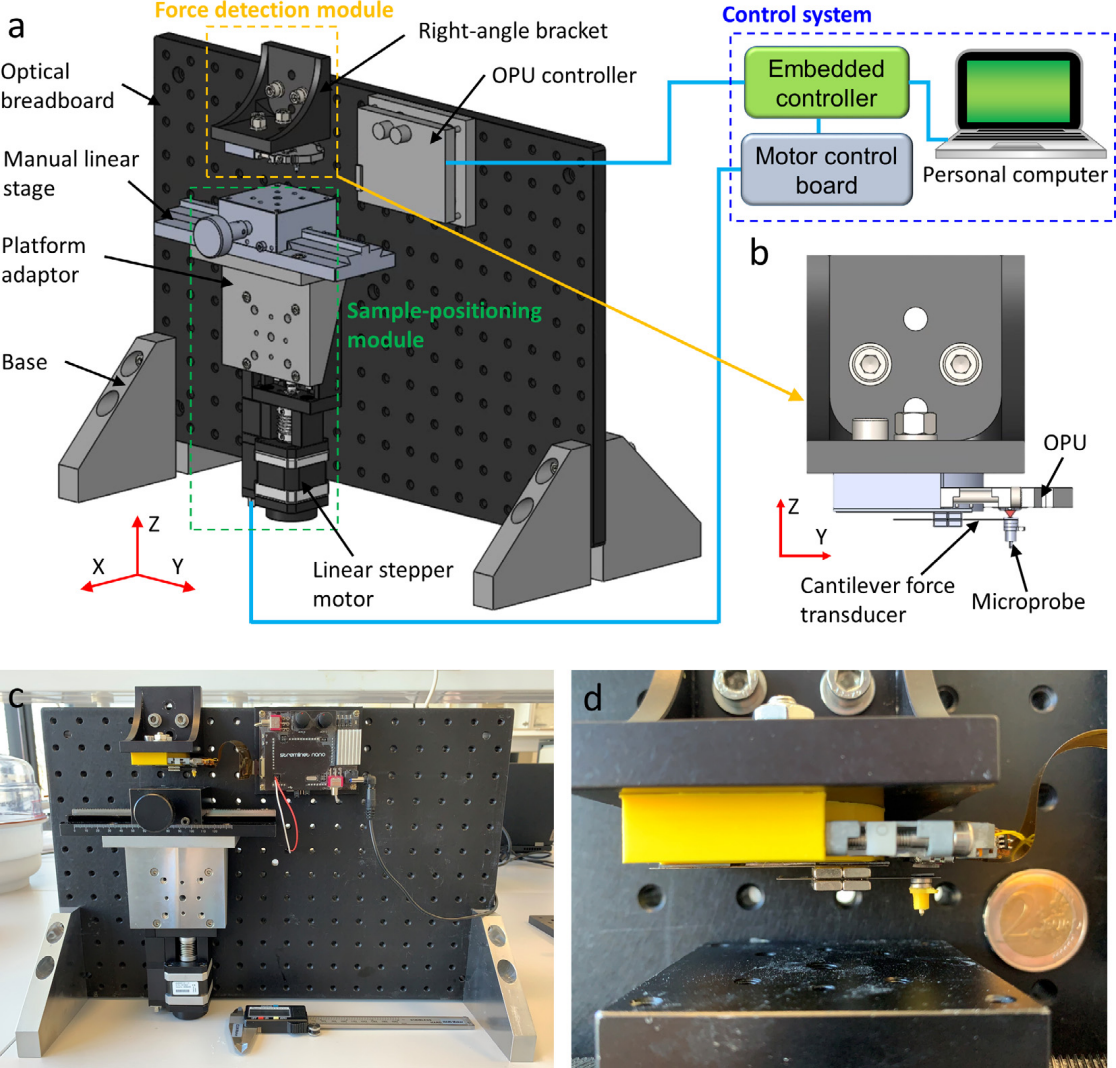
Development of a force analyzer using a DVD optical-pickup-unit (OPU) to detect the cantilever force transducer with the applied force from Newton to nano-Newton. **a)** Diagram of the force analyzer, including force detection and sample-positioning modules and a control system. **b)** Detailed diagram of force detection module assembled by an OPU, a cantilever force transducer, and a microprobe. **c)** Photograph of the OPU force analyzer. **d)** Detailed photograph of force detection module.

In this study, we custom-built an OPU force analyzer by repurposing the use of a DVD OPU to achieve a force-sensing range from Newton to nano-Newton (Fig. 1). In addition, we implemented different cantilever-based force transducers to easily fit the target force-sensing ranges. The proposed OPU force analyzer provides an open-source design and fulfills the force-sensing gap between the currently available mucoadhesion force measurement methods.

## Hardware description

This OPU force analyzer mainly contains a force detection module, a sample-positioning module, and a control system (Fig. 1**a and c**). While the sample-positioning module approaches or withdraws a sample to a microprobe, the force detection module senses the applied force (Fig. 1**b and d**). Simultaneously, the control system controls the movement of the sample-positioning module and receives the sensing signal from the force detection module.

### Force detection module

**Fig. 2:**
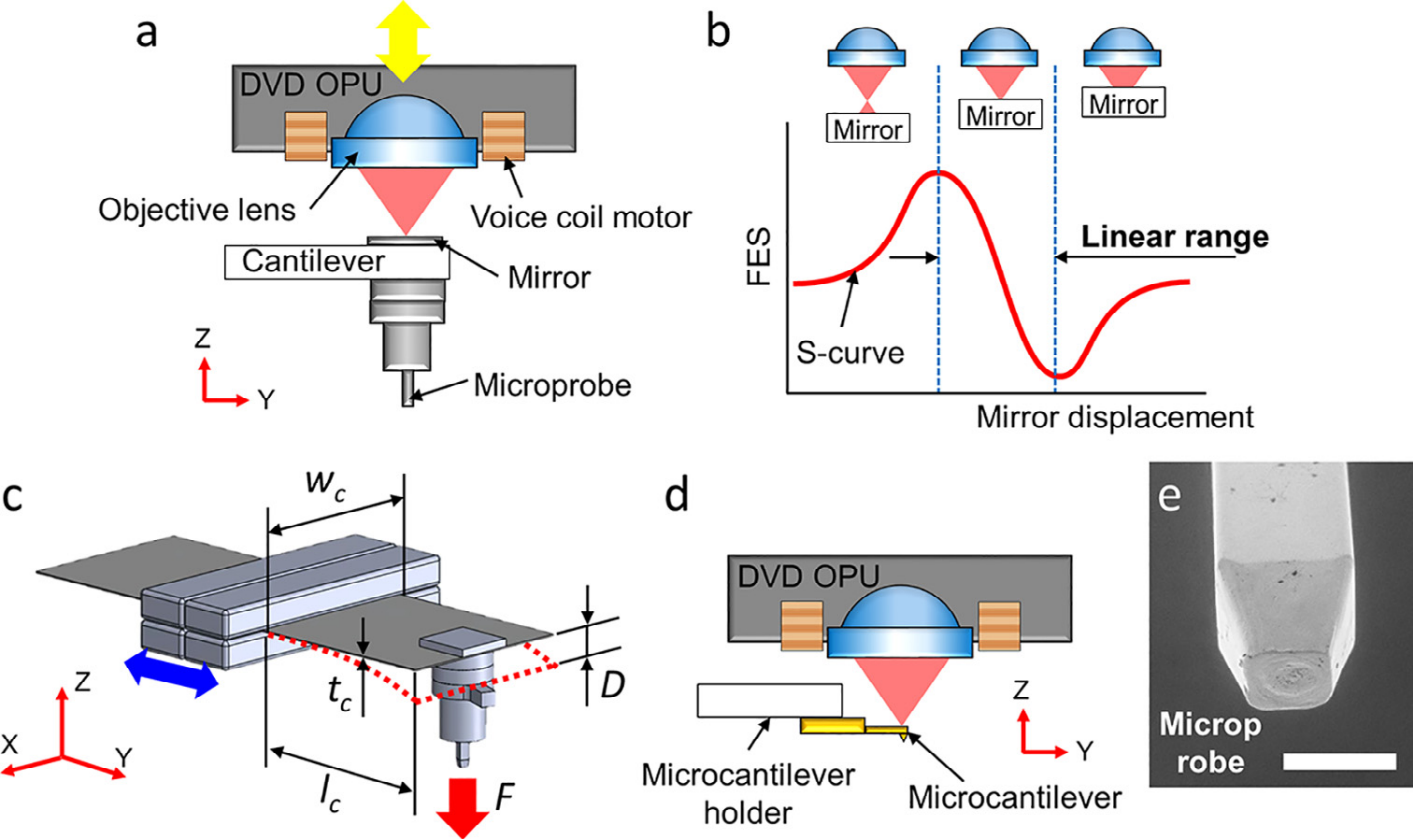
Illustration of the force detection module. **a)** The DVD OPU focuses a 650-nm-wavelength laser beam on a mirror to sense the deflection of the cantilever force transducer. **b)** Focus error signal (FES) presents an S-curve, while displacing the mirror along the z-axis direction. The S-curve presents a highly sensitive linear range. **c)** The cantilever deflection *D* caused by the applied force *F*. The force detecting range of the cantilever force transducer is defined by the thickness (*t_c_*) and length (*l_c_*) of the cantilever. The length of the cantilever is modified by shifting four rectangle magnets along the y-axis direction. **d)** Installation of microcantilevers for nano-Newton scale force measurement. **e)** SEM image of the microprobe (scale bar represents 500 µm).

Fig. 2**a** shows the force detection module consisting of the OPU and the cantilever-based force transducer. The OPU emits a laser with a wavelength of 650 nm, and a built-in voice coil motor actuates an objective lens in the z-axis direction to precisely focus the laser on the mirror. Furthermore, the OPU integrates an astigmatic optical path and a sensor to generate a focus error signal (FES) [13]. While the mirror approaches along the z-axis to a laser focal point, the FES appears as an S-shaped curve (S-curve) (Fig. 2**b**). The middle part of the S-curve represents a highly sensitive linear signal that monitors the cantilever deflection with a nanometer-scale resolution. Thus, the FES can detect the cantilever deflection caused by an applied force. The DVD OPU has an FES linear range of approximately a few microns.

**Fig. 3:**
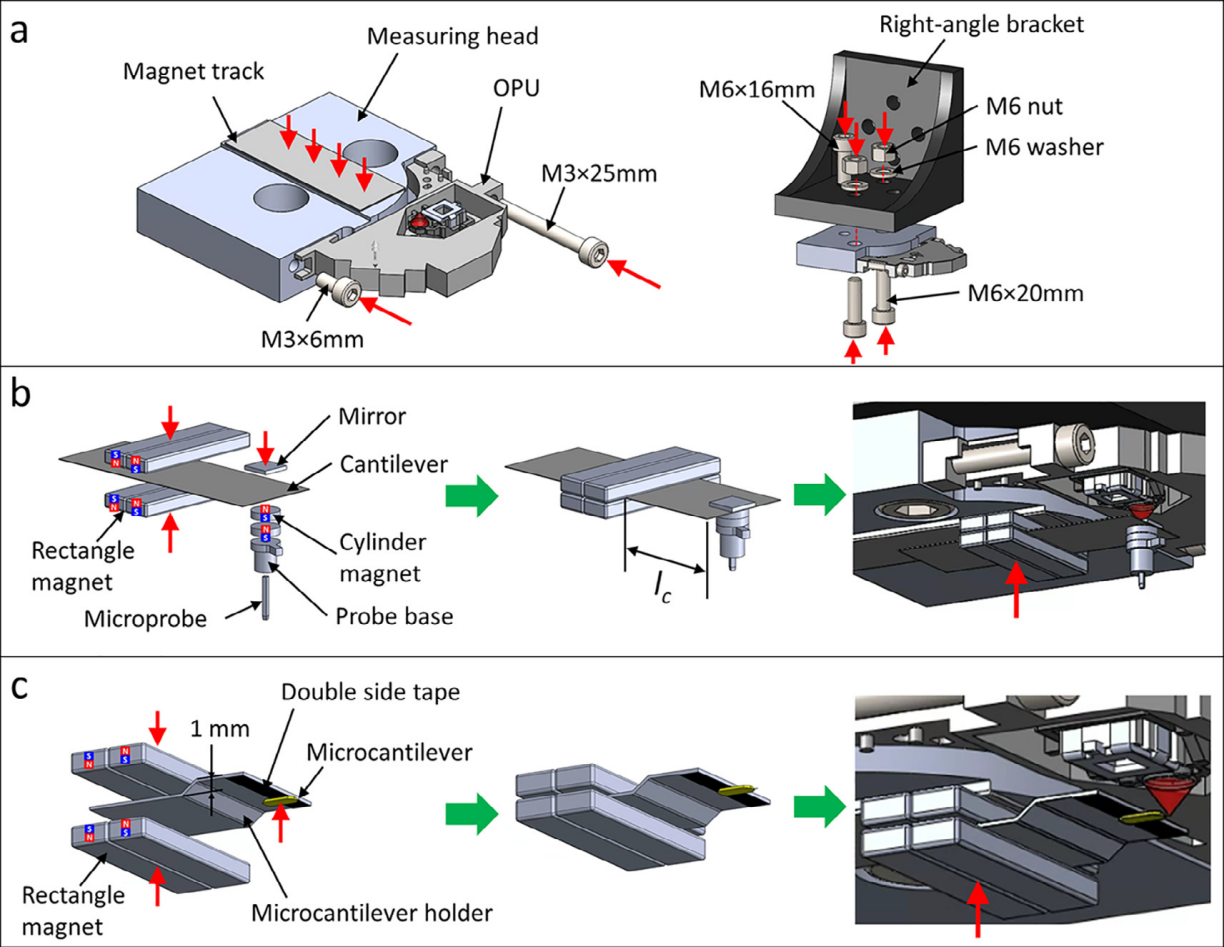
Assembly of **a)** force detection module, **b)** cantilever force transducer, and **c)** force transducer equipped with microcantilever.

**Fig. 4:**
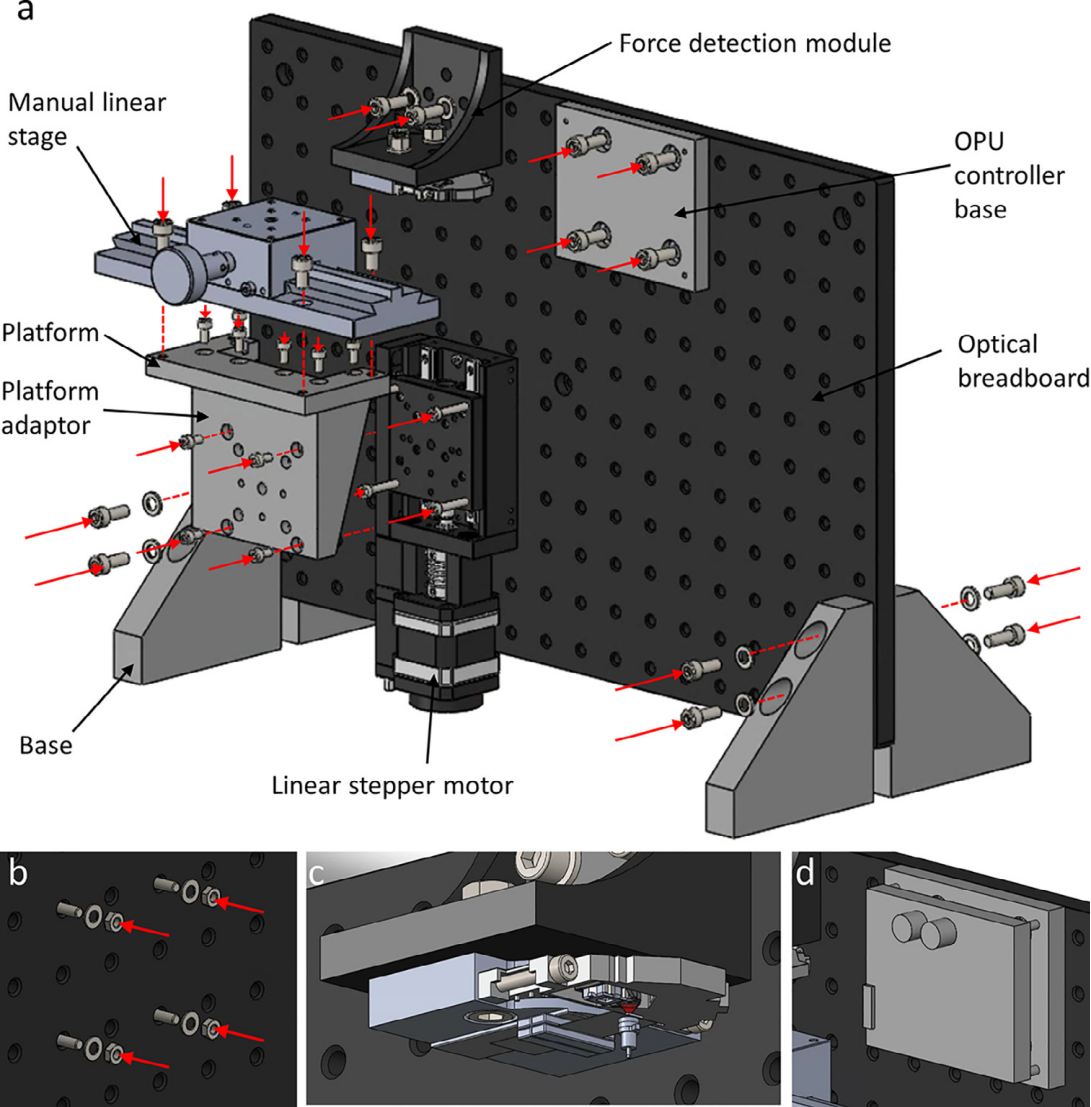
Assembly diagram: **a)** the whole system, **b)** fixing the linear stepper motor (view from the backside of optical breadboard), **c)** fixing the force detection module with the cantilever force transducer, and **d)** fixing the OPU controller.

**Fig. 5:**
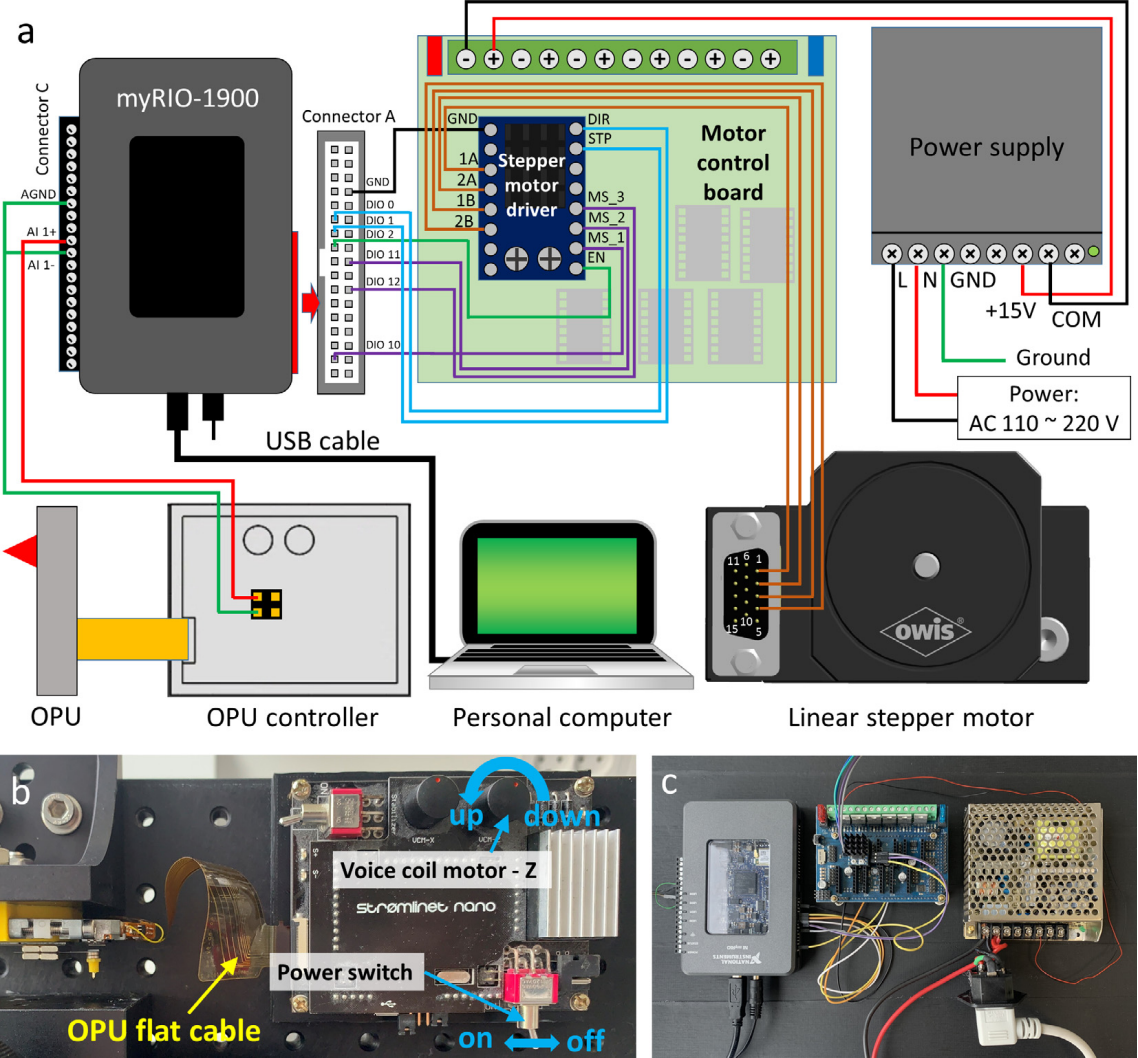
Illustration of the controlling system of the OPU force analyzer. **a)** Wiring diagram. **b)** Photograph of the OPU connection and controller operation. **c)** Photograph of the controlling system.

**Fig. 6:**
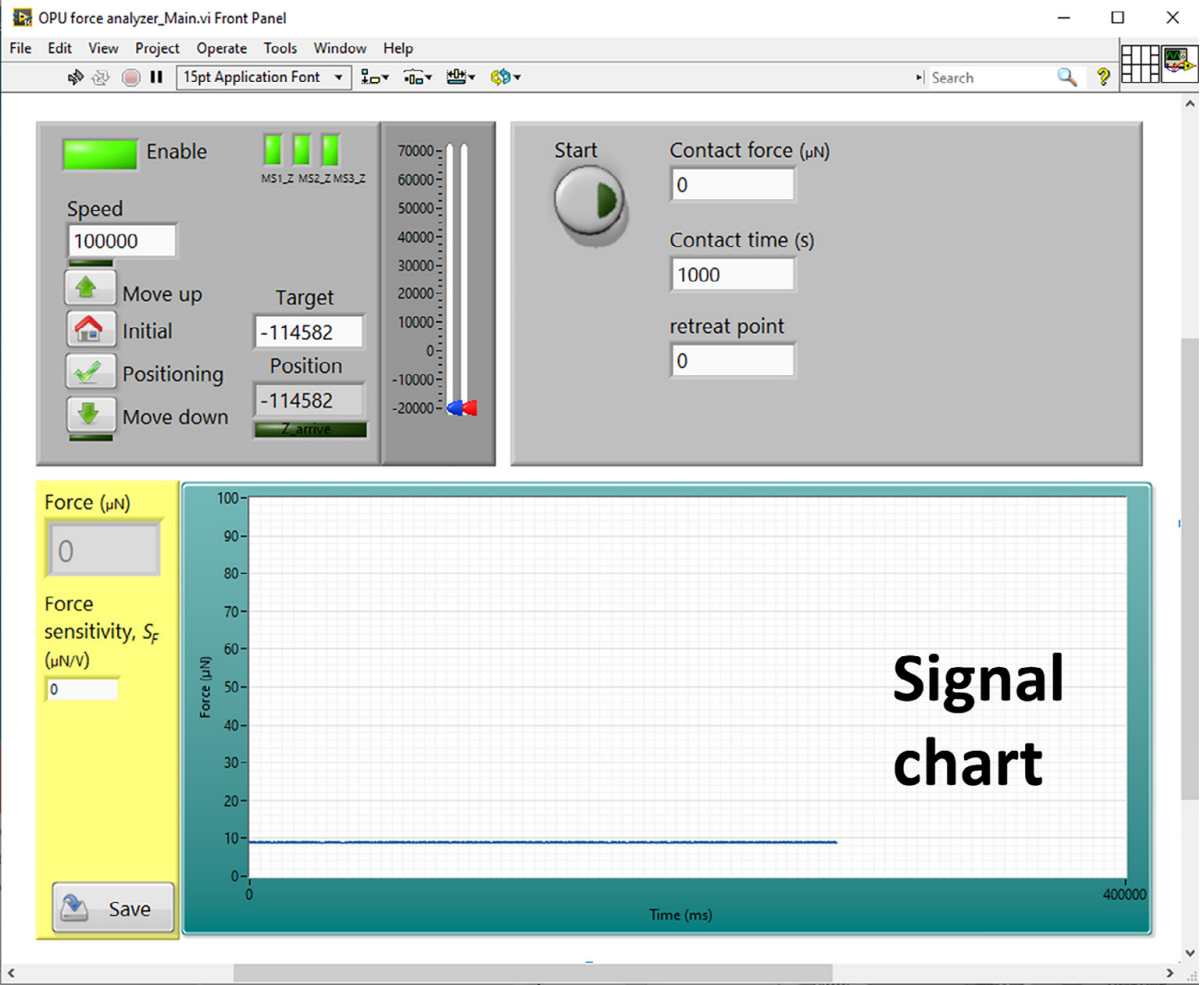
The interface of the operation program for implementing force measurement, while setting parameters of contact force, contact time, retreat point, speed, and force sensitivity.

**Fig. 7:**
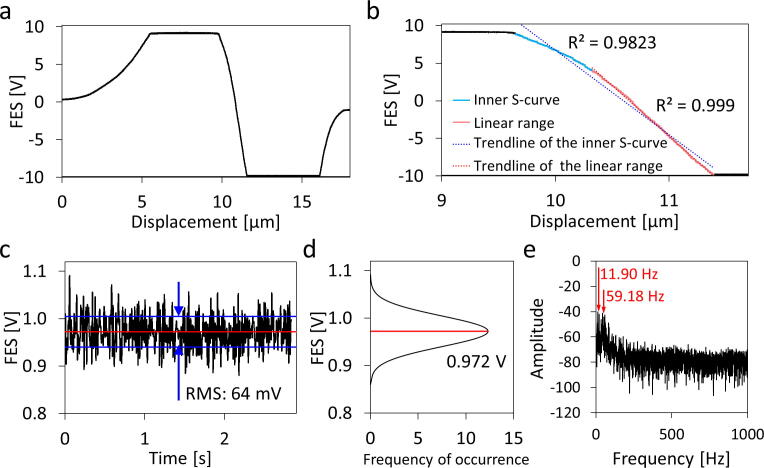
S-curve linear range and noise analysis. **a)** The whole S-curve detected from 8.68 to −9.98. V. **b)** Analysis of the inner S-curve showing that the signal between 3.76 and −9.98 V appears in a linear region, as the R-squared value is 0.999. **c)** Signal noise of FES in the linear region of the S-curve shows an RMS noise of 64 mV, after processed by a 100 Hz low-pass filter. **d)** The signal noise shows a Gaussian distribution. e) Signal noise analysis by a fast Fourier transform indicated an 11.90 Hz and a 59.18 Hz noise in the spectrum.

**Fig. 8:**
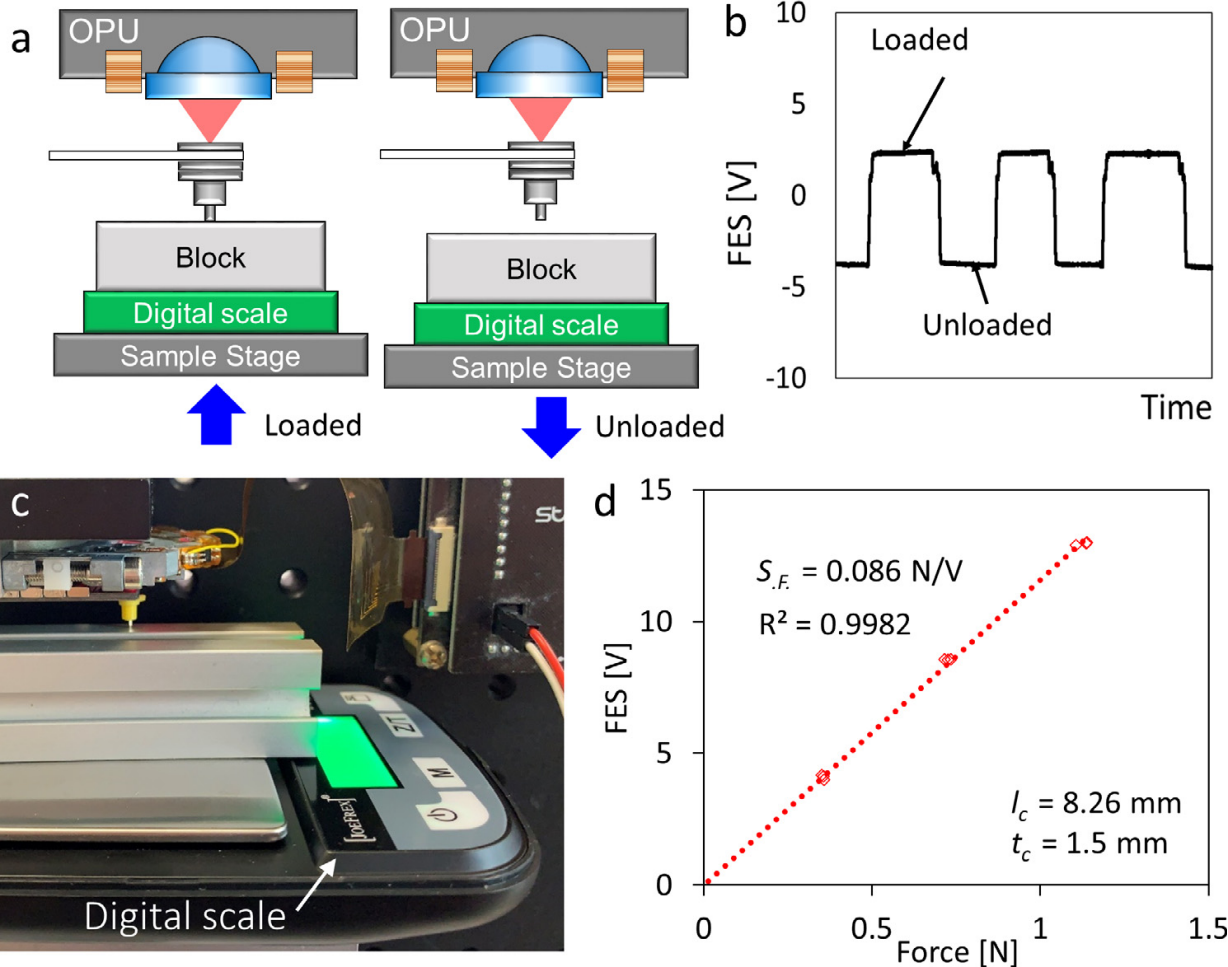
Force calibration of the cantilever force transducer ranging from Newton to milli-Newton. **a)** Diagram of the calibration process showing that the OPU monitors the cantilever deflection while the sample stage positions the digital scale upward to load the microprobe and downward to unload the microprobe. **b)** Focus error signal (FES) step signal while loading/unloading the cantilever force transducer. **c)** Photograph of the force calibration process. **d)** Calibration curve using a 1.5 mm-thick cantilever with a force sensitivity (*S_.F._*) of 0.086 N/V, while applying a force ranging from 1.1 N to 5.50 mN, n = 3, the standard residual error is 0.167 V.

**Fig. 9:**
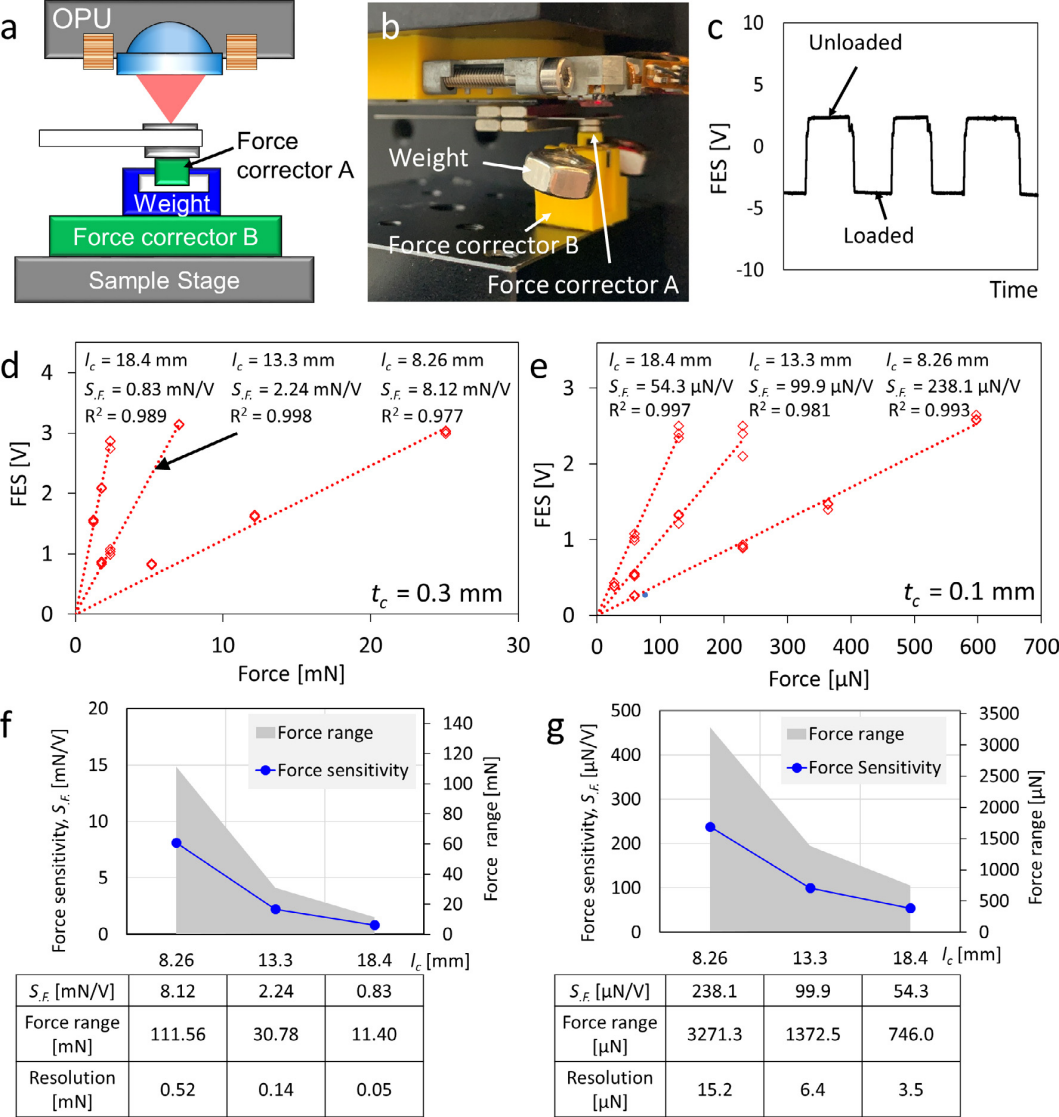
Force calibration of the cantilever force transducer in the range from milli- to micro-Newton **a)** Diagram of the calibration process by loading a known weight on the cantilever force transducer and unloading the cantilever by lifting the sample stage. **b)** Photograph of the force calibration process. **c)** Focus error signal (FES) step signal while loading/unloading the cantilever force transducer. **d)** Calibration curves of milli-Newton scale force using a 0.3 mm-thick cantilever with force sensitivity (*S_.F._*) of 0.83, 2.24, and 8.12 mN/V, with adjusted cantilever length of 18.4, 13.3, and 8.26 mm, respectively (n = 3, standard residual error: 0.060, 0.044 and 0.842 V.) **e)** Calibration curves of milli-Newton scale force using a 0.1 mm-thick cantilever with force sensitivity (*S_.F._*) of 54.3, 99.9, and 238.1 µN/V, with adjusted cantilever length of 18.4, 13.3, and 8.26 mm, respectively (n = 3, standard residual error: 0.051, 0.117, 0.073 V.) Force sensitivity (*S_.F._*), force range, and resolution of each cantilever parameter setting of **f)** milli-Newton range and **g)** micro-Newton range.

**Fig. 10:**
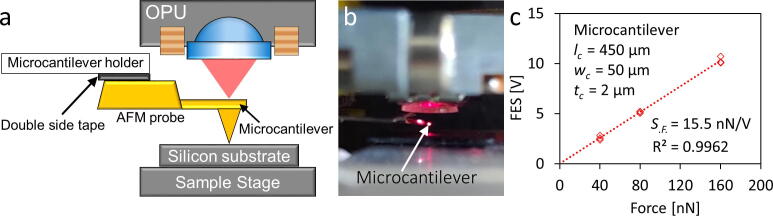
Force sensitivity estimation of nano-Newton force transducer. **a)** Diagram of the estimation process showing that the OPU monitors the microcantilever deflection induced by the movement of the sample stage. The force constant of the microcantilever is 0.2 nN/nm. **b)** Photograph of the force sensitivity estimation process. **c)** Estimation curve of nano-Newton scale using a microcantilever presenting a force sensitivity (*S_.F._*) of 15.5 nN/V, while the sample stage moves 200, 400 and 800 nm upward, corresponding to applied force of 40, 80, and 160 nN, respectively, n = 3, the standard residual error is 0.225 V.

The cantilever-based force transducer converts the applied force to a structural deflection during the measurement process (Fig. 2**c**). The applied force *F* is proportional to the cantilever deflection *D*, as described in the following formulation [20]:

(1) $$F=D∙E_{c}w_{c}{t_{c}}^{3}/4{l_{c}}^{3}$$

where *E_c_* is the Young's modulus of the cantilever, *and w_c_*, *t_c_*, and *l_c_* are the width, thickness, and length of the cantilever, respectively.

The force detection module utilizes different cantilever-based force transducers to detect the applied forces from Newton to micro-Newton scale. Steel shim-based cantilevers can easily be adjusted to fit a suitable measuring force range by selecting the shim thickness and/or adjusting the cantilever length, according to **Equation** (1). In addition, a commercial microcantilever (AFM probe) can be directly attached to the edge of the steel shim to detect the force on the nano-Newton scale (Fig. 2**d**).

The force detection module utilizes a microprobe which is a straight pin, an off-the-shelf, standardized electronic component (Fig. 2**e**). Compared to the centimeter-scale probe on a commercial texture analyzer, this microprobe is suitable for carrying a single micrometer-scale device. Furthermore, within the microscale contact area, the microprobe can increase the reproducibility of the measurements of the micro-Newton mucoadhesion force. Since a fresh mucus tissue has a topography variation in the millimeter range, the centimeter-scale probe cannot uniformly contact the mucus layer. Minimizing the contact area of the probe will reduce the variation from the mucus layer surface, which decreases the uncertainty of contact conditions and increases the reproducibility of the measurements.

### Sample-positioning module

A manual linear stage carries the sample, allowing the adjustment of the sample position along the y-axis (Fig. 1**a**). To approach and withdraw the sample to the microprobe, we used a linear stepper motor (LTM 60–25, OWIS GmbH, Staufen, Germany) that provides a total travel distance of 25 mm along the z-axis. The stepper motor moves 5 µm per step. By a maximum of 128 divided stepper motor drivers, the stage reaches the highest positioning resolution of 40 nm for a single sub-step. Within the highest positioning resolution setting, the motor has a maximum positioning speed of 1.56 mm/s.

### Control system

An embedded controller (myRIO-1900; National Instruments, Austin, TX, USA) is connected to an OPU controller and a motor control board (Fig. 1**a**). The OPU controller calculates the FES and drives the voice coil motor. The motor control board controls the stepper motor for the sample positioning. The embedded controller communicates with a computer through a graphical user interface program to control the force measurement process.

In summary, the presented OPU force analyzer offers the following advantages:

• Nanometer-scale sensitivity and a broad force measuring range from Newton to nano-Newton.
• Increased reproducibility for mucoadhesion force characterization of microscale devices by the microprobe.
• Cost-effective components and an open-source design provide users with easy customization for various force measurement tasks, such as indention and compression tests.

## Design files

The design files were drawn using computer-aided design (CAD) software SolidWorks 2014 (Dassault Systèmes SolidWorks Corporation, Massachusetts, USA). All the design files can be downloaded from the linked Mendeley data repository.

### CAD files

**Table:** 

Design file name	File type	Open source license	Location of the file
Base.SLDPRT	CAD	CC BY 4.0	https://doi.org/10.17632/cnkd95kp65.1
Measuring head.SLDPRT	CAD	CC BY 4.0	https://doi.org/10.17632/cnkd95kp65.1
Platform.SLDPRT	CAD	CC BY 4.0	https://doi.org/10.17632/cnkd95kp65.1
Platform adaptor.SLDPRT	CAD	CC BY 4.0	https://doi.org/10.17632/cnkd95kp65.1
Probe base.SLDPRT	CAD	CC BY 4.0	https://doi.org/10.17632/cnkd95kp65.1
Force corrector A.SLDPRT	CAD	CC BY 4.0	https://doi.org/10.17632/cnkd95kp65.1
Force corrector B.SLDPRT	CAD	CC BY 4.0	https://doi.org/10.17632/cnkd95kp65.1
OPU controller base.SLDPRT	CAD	CC BY 4.0	https://doi.org/10.17632/cnkd95kp65.1
Microprobe.SLDPRT	CAD	CC BY 4.0	https://doi.org/10.17632/cnkd95kp65.1
Cantilever.SLDPRT	CAD	CC BY 4.0	https://doi.org/10.17632/cnkd95kp65.1
Magnet track.SLDPRT	CAD	CC BY 4.0	https://doi.org/10.17632/cnkd95kp65.1
Microcantilever holder.SLDPRT	CAD	CC BY 4.0	https://doi.org/10.17632/cnkd95kp65.1

### 3D printing files

All 3D printing files are available in stereolithography (STL) format on the Mendeley data repository. All parts were printed by a fused deposition 3D printer, Prusa i3 MK2.5S printer (Prusa Research, Prague, Czech Republic) with a 0.4 mm nozzle and a PLA filament with a layer height of 0.1 mm and 30 % infill rate.

**Table:** 

Design file name	Number of required prints	Open source license	Location of the file
Measuring head.stl	1	CC BY 4.0	https://doi.org/10.17632/cnkd95kp65.1
Probe base.stl	1	CC BY 4.0	https://doi.org/10.17632/cnkd95kp65.1
Force corrector A.stl	1	CC BY 4.0	https://doi.org/10.17632/cnkd95kp65.1
Force corrector B.stl	1	CC BY 4.0	https://doi.org/10.17632/cnkd95kp65.1
OPU controller base.stl	1	CC BY 4.0	https://doi.org/10.17632/cnkd95kp65.1

### Software and firmware

The firmware and the operation program were edited and compiled using the LabVIEW 2016 software (National Instruments, Austin, TX, USA). A compressed file containing all the programs is available in the Mendeley data repository listed in the following table. All unzipped program files are located in the same folder to maintain the original data path.

**Table:** 

Design file name	File type	Open source license	Location of the file
OPU force analyzer	Zip	CC BY 4.0	https://doi.org/10.17632/cnkd95kp65.1

## Bill of materials

The bill of materials includes mechanical mechanisms, electronic components, controllers, and other needed components. The prices vary from different suppliers or source channels. The displayed costs in the table match the price at the moment of purchase. The current prices can be found in the listed source.

**Table:** 

Designator	Component	Number	Cost per unit [EUR]	Total cost [EUR]	Source of materials	Material type
Base	Base	4	50	200	CNC workshop	Metal
Platform	Platform	1	50	50	CNC workshop	Metal
Platform adaptor	Platform adaptor	1	67.75	67.75	CNC workshop	Metal
OPU	DVD OPU Top1300s	1	6.26	6.26	Amazon	Electronics
OPU controller	OPU controller	1	460.69	460.69	Stromlinet Nano	Electronics
Steel shim 0.1	Steel shim tape T0.1 W12.7L1000	1	6.51	6.51	Misumi	Metal
Steel shim 0.3	Steel shim tape T0.3 W12.7L1000	1	6.51	6.51	Misumi	Metal
Mirror	Polished SI wafer	1	18.7	18.7	Amazon	Silicon
Rectangle magnet	Rectangle magnet L20W4T2	4	0.2	0.8	Amazon or Supermagnete	Metal
Cylinder magnet	Cylinder magnet D4L1 (NO118)	10	1.635	16.35	Misumi	Metal
Linear stepper motor	Linear stepper motor (LTM 60–25)	1	1029	1029	Owis	Metal
Manual linear stage	Manual linear stage (One Knob Stage & 125 mm Track Combination Stock #59–263)	1	194	194	Edmund	Metal
Optical breadboard	Optical Breadboard (MB3045_M)	1	181.3	181.3	Thorlabs	Metal
Right-angle bracket	Right-Angle Bracket (MT402)	1	49.68	49.68	Thorlabs	Metal
Power supplier	Power supplier 15 V	1	21.43	21.43	RS Components	Electronics
myRIO-1900	Embedded controller (myRIO-1900)	1	640	640	National instrument	Electronics
Stepper motor driver	Stepper motor driver (RAPS 128 Stepper Driver)	1	17.69	17.69	Reprap.me	Electronics
Motor control board	Motor control board (RADDS v1.6 board)	1	57.49	57.49	Reprap.me	Electronics
Straight pin	Straight Pin (HARWIN M20, 2.54 mm Pitch, 36 Way, 1 Row, Straight Pin Header, Through Hole)	1	1.22	0.83	RS Components	Electronics
M6×20mm screw	M6×20 mm Hex head cap screws (SH6MS20)	2	0.342	0.684	Thorlabs	Metal
M6×16mm screw	M6×16 mm Hex head cap screws (SH6MS16)	15	0.322	4.836	Thorlabs	Metal
M6×10mm screw	M6×10 mm Hex head cap screws(SH6MS10)	4	0.300	1.203	Thorlabs	Metal
M4×25mm screw	M4×25 mm Hex head cap screws(SH4MS25)	4	0.150	0.601	Thorlabs	Metal
M4×8mm screw	M4×8 mm Hex head cap screws(SH4MS8)	10	0.123	1.23	Thorlabs	Metal
M3×25mm screw	M3×25 mm Hex head cap screws	1	0.20	0.20	Amazon or hardware store	Metal
M3×6mm screw	M3×6 mm Hex head cap screws (SH3M06)	5	0.145	0.725	Thorlabs	Metal
M6 nut	M6 nut	2	0.15	0.3	Amazon or hardware store	Metal
M4 nut	M4 nut	4	0.10	0.40	Amazon or hardware store	Metal
M6 washer	M6 washer (W25S050)	12	0.045	0.541	Thorlabs	Metal
M4 washer	M4 washer (W8S038)	4	0.0325	0.13	Thorlabs	Metal
PLA	PLA polymer	1	17.15	17.15	Reprap.me	Polymer
Microcantilever	Microcantilever (PPP-CONT)	1	27.6	27.6	Nanoandmore Gmbh	Silicon

## Build Instructions

The assembly process of the mucoadhesion force analyzer requires instant glue and double-sided tape to firmly bond the components. The screw holes of the 3D printed parts are designed for a direct screw-in fixture; therefore, hole thread tapping is not required. One needs to be aware of all the required parts of 3D printing before the assembly process.

### Force detection module assembly (Fig. 3**a**)

1. Components needed: 1 × steel shim 0.3, 1 × OPU, 1 × measuring head, 1 × right-angle bracket, 2 × M6×20mm screws, 1 × M6×16mm screw, 1 × M3×25mm screw, 1 × M3×6mm screw, 2 × M6 nuts, and 2 × M6 washers.
2. Cut 38 mm of the steel shim 0.3 as a magnet track.
3. Glue the magnet track on the measuring head.
4. Fix the OPU on the measuring head with an M3×25mm screw and an M3×6mm screw.
5. Fix the measuring head on the right-angle bracket with an M6×16mm screw, two M6×20mm screws, two M6 nuts, and two M6 washers.

### Cantilever force transducer assembly (Fig. 3**b**)

1. Components needed: 1 × steel shim 0.1, 4 × rectangle magnets, 2 × cylinder magnet, 1 × straight pin, 1 × probe base, and 1 × mirror.
2. Cut 35 mm of the steel shim 0.1 as a cantilever force transducer.
3. Glue a mirror on the edge of the cantilever
4. Below the mirror, glue a cylinder magnet on the bottom side of the cantilever.
5. Fix the cantilever to a target length (*l_c_*) with four rectangle magnets.
6. Cut 10 mm of the straight pin as a microprobe.
7. Glue the microprobe in the cavity of the probe base, avoiding the protrusion of the microprobe from the backside.
8. Glue a cylinder magnet on top of the probe base.
9. Place the cantilever force transducer on the magnet track and align the mirror to the OPU laser focal spot area.
10. To prepare different thickness cantilevers, repeat the assembly process 2–5.
11. The cantilever length and thickness influence the force measurement range. The force calibration for the reference is presented in Section 7.2.

### Microcantilever force transducer assembly (Fig. 3**c**)

1. Components needed: 1 × steel shim 0.3, 4 × rectangle magnets, and 1 × microcantilever.
2. Cut 20 mm of the steel shim 0.3.
3. Bend it as a microcantilever holder (Fig. 3**c**).
4. Fix a microcantilever on the edge of the microcantilever holder with double-sided tape.
5. Fix the cantilever with four rectangle magnets.
6. Place the microcantilever holder on the magnet track and align the microcantilever to the laser focal spot area.

### Assembly of the whole system (Fig. 4)

1. Components needed: 1 × optical breadboard, 4 × bases, 1 × OPU controller base, 1 × linear stepper motor, 1 × platform, 1 × platform adaptor, 1 × manual linear stage, 1 × force detection module, 1 × OPU controller, 14 × M6×16mm screws, 4 × M6×10mm screws, 4 × M4×25mm screws, 10 × M4×8mm screws, 4 × M3×6mm screws, 10 × M6 washers, 4 × M4 washers, and 4 × M4 nuts.
2. Fix four bases on the four corners of the optical breadboard with eight M6×16mm screws (Fig. 4**a**). Slightly adjust the vertical position of each base to allow the system to stably stand on a flat surface.
3. Fix the OPU controller base on the optical breadboard with four M6×16 mm screws.
4. Fix the linear stepper motor on the optical breadboard with four M4×25mm screws, four M4 nuts, and four M4 washers (Fig. 4**a and b**).
5. Fix the platform adaptor on the platform of the linear stepper motor with four M4×8mm screws.
6. Fix the platform on the platform adaptor with six M4×8mm screws.
7. Fix the manual linear stage on the platform with four M6×10mm screws.
8. Fix the force detection module on the optical breadboard with two M6×16mm screws and two M6 washers (Fig. 4**a and c**).
9. Fix the OPU controller on the OPU controller base with four M3×6mm screws (Fig. 4**d**).

### Wiring the control system (Fig. 5)

1. Components needed: 1 × myRIO-1900, 1 × OPU controller, 1 × OPU, 1 × stepper motor driver, 1 × motor control board, 1 × linear stepper motor, and 1 × power supply.
2. Connect the OPU controller's FES output pins to the myRIO-1900 ports A1 + and AGND (connector C). Then connect myRIO-1900 ports A1- and AGND (Fig. 5**a**).
3. Connect the OPU flat cable to the OPU controller (Fig. 5**b**).
4. Mount the stepper motor driver on the motor control board (Fig. 5**a and c**).
5. Connect the stepper motor driver's pins DIR, STP, MS_3, MS_2, MS_1, EN, and GND to ports DIO1, DIO0, DIO12, DIO11, DIO10, DIO2, and GND (myRIO-1900 connector A), respectively.
6. Connect the stepper motor driver's pins 1A, 2A, 1B, and 2B to the linear stepper motor's pins 1, 2, 3, and 4, respectively.
7. Connect the power supply to the OPU controller.
8. Connect the power supply to 110 – 220 V and the OPU controller to the power adaptor.
9. Connect myRIO-1900 to a computer.

## Operation Instructions (Fig. 6)

1. Switch on the myRIO-1900, the power supply, and the OPU controller power (Fig. 5**b**).
2. Open a LabVIEW project named “OPU force analyzer” and a Vi named “OPU force analyzer.” Then, press the “run” button to start the program. The FES appears in the signal chart.
3. Adjust a voice coil motor-z knob (on the OPU controller, Fig. 5**b**) clockwise/counterclockwise to shift the objective lens upward/downward. Utilize the knob to adjust the vertical position of the laser spot.
4. Focus the laser spot directly on the mirror on the top side of the cantilever force transducer (Fig. 2**a**) or the microcantilever (Fig. 2**d**).
5. Adjust the knob and shift the FES to the center of the linear range (Fig. 2**b**).
6. Place a sample on the manual linear stage.
7. Activate the linear stepper motor by clicking the “Enable” button, and move the sample to a suitable position using the “move up” and “move down” buttons.
8. Set the measurement parameters, “contact force,” “contact time,” “retreat point,” and “speed.” The retreat point represents the stop position while withdrawing the sample from the microprobe.
9. Set the “force sensitivity (*S_.F._*)” to convert the FES voltage to a force reading value. The *S_.F._* can be referred to in Section 7.2 calibration process.
10. Click the “start” button to enable the measurement procedure. The signal chart plots and records the measured force curves.
11. After the measuring process, press the “save” button to save the recorded signal chart.

## Validation and characterization

### Linear range and noise analysis (Fig. 7)

It is crucial to characterize the linear range of the S-curve and the FES noise level before sensing the cantilever-based force transducer. Fig. 7**a** shows the S-curve with the upper and lower signal cutoffs (due to signal saturation). The S-curve linear range was further analyzed, as shown in Fig. 7**b**. The full range inner S-curve (blue line, 8.68 to −9.98 V) has an R-squared value of 0.982, which is unsuitable for an accurate displacement measurement. When reducing the sensing range from 3.76 to −9.98 V, the R-squared value is 0.999, showing much better signal linearity. Thus, the OPU force analyzer is working at this linear range (red line) with an effective displacement sensing range of 1.07 µm and a sensing sensitivity of 0.077 nm/mV.

The FES from the OPU controller has a root mean squared (RMS) noise level of 28 mV, which corresponds to a displacement sensing noise of 2.2 nm. Further, we investigated the combined system noise level by focusing the focal spot on the mirror on the top side of the cantilever. Under these conditions, the FES signal containing environmental vibration noise and electrical noise from the OPU controller was processed by a 100 Hz low-pass filter (Fig. 7**c**). Hence, the FES used for detection has an RMS noise of 64 mV, corresponding to a displacement sensing noise of 4.9 nm. A Gaussian distribution statistical analysis (Fig. 7**d**) shows that the FES signal noise has a mean value and standard deviation of 0.972 V and 0.032 V, respectively. Furthermore, a fast Fourier transform of the signal shows an 11.90 Hz and a 59.18 Hz noise caused by environmental vibration and electrical noise, respectively (Fig. 7**e**). The combined system noise could be reduced by further improvements, such as strengthening the system rigidity, isolating environmental vibration, and shielding sensitive signals.

### Force transducers calibration and estimation

After the signal sensitivity characterization, the OPU force analyzer can monitor different cantilever-based force transducers to reach different target force measurement ranges.

#### Newton to milli-Newton force transducer calibration **(**Fig. 8**)**

To achieve a force-sensing range from the Newton to milli-Newton scale, five 0.3 mm steel shims were stacked and glued into a 1.5 mm-thick cantilever transducer. The length of the cantilever was adjusted to 8.26 mm. The microprobe was attached to the free end of the thick cantilever. A digital scale (JoeFrex Corp., Huston, United States) at the sample stage was driven upward to contact the microprobe. The digital scale was calibrated by a precision balance (see Appendix A. Supplementary material Figure S1). Three contact force (translated from the weight) values from the digital scale were used to calibrate the FES, with a force sensitivity (*S_.F._*) of 0.086 N/V. The thick cantilever transducer enables the OPU force analyzer to measure a force ranging from 1.1 N to 5.50 mN.

#### Milli-Newton to micro-Newton force transducers calibration **(**Fig. 9**)**

A different method was used to calibrate the cantilever force transducers for the milli-Newton and micro-Newton ranges. A standard steel shim (*t_c_* = 0.3 mm) was used as a milli-Newton cantilever transducer. Fig. 9**a** shows a force corrector A attached to the bottom of the cantilever. Different known weights from 2559.8 to 122.9 mg (weighted by precision balance XPE26, Mettler Toledo, Greifensee, Switzerland) were loaded on the force corrector A (Fig. 9**b**). The force corrector B on the sample stage was driven upward, and the weight was unloaded. The weight loading and unloading processes were monitored by the FES (Fig. 9**c**), which was then calibrated with multiple cantilever lengths (*l_c_* = 8.26, 13.3, and 18.4 mm) settings (Fig. 9**d**). A micro-Newton cantilever transducer is a thin steel shim (*t_c_* = 0.1 mm) that is calibrated using the same process with the known weights from 60.98 to 2.74 mg (Fig. 9**e**). Both the milli-Newton and micro-Newton cantilever transducers have linear correspondences between the FES and weight at different *l_c_* length settings.

The milli- and micro-Newton cantilever transducers are primarily used for single microdevice mucoadhesion force measurements, and therefore, we further analyzed the force-sensing ranges and sensitivities of both transducers with different cantilever length settings. Fig. 9**f** shows the force-sensing range, sensitivity, and resolution of the milli-Newton transducer, which can sense a maximum force of 111.56 mN and the highest resolution of 0.05 mN. The micro-Newton transducer can reach a maximum force limit of 3,271.3 µN and the highest resolution of 3.5 µN (Fig. 9**g**). Users can reference the above-mentioned *l_c_ and t_c_* parameters to find a suitable force-sensing range for their experiments. Besides, this measurement system performs a reproducibility of 1.42 µN for measuring the adhesion force of a single microdevice in the micro-Newton force range (see Appendix A. Supplementary material Table S1).

#### Nano-Newton force transducer sensitivity estimation **(**Fig. 10**)**

For nano-Newton range force sensing, we utilized an AFM probe (PPP-CONT, Nanosensors^TM^, Neuchatel, Switzerland) containing a microcantilever with an *l_c_* = 450 µm, *w_c_* = 50 µm, and *t_c_* = 2 µm, and a tip (height = 15 µm) at the bottom side of the microcantilever. The nanoscale laser spot of the OPU is beneficial for monitoring the microcantilever, which can function as a nano-Newton force transducer. The AFM probe was attached to the microcantilever holder using double-sided tape (Fig. 10**a and b**). The OPU laser can directly focus on the microcantilever (Section 6. “Operation Instructions” step 4 to align the laser focal spot). A silicon substrate on the sample stage was positioned upward and touched the tip during the process. The stage further moved 200, 400, and 800 nm, while the FES monitored the microcantilever deflection simultaneously. Since the microcantilever has a typical force constant of 0.2 nN/nm, the OPU force analyzer can reach an estimated force sensitivity of 15.5 nN/V and a resolution of 0.99 nN (Fig. 10**c**).

### Preliminary study of mucoadhesion force alteration while time and moisture escape

**Fig. 11:**
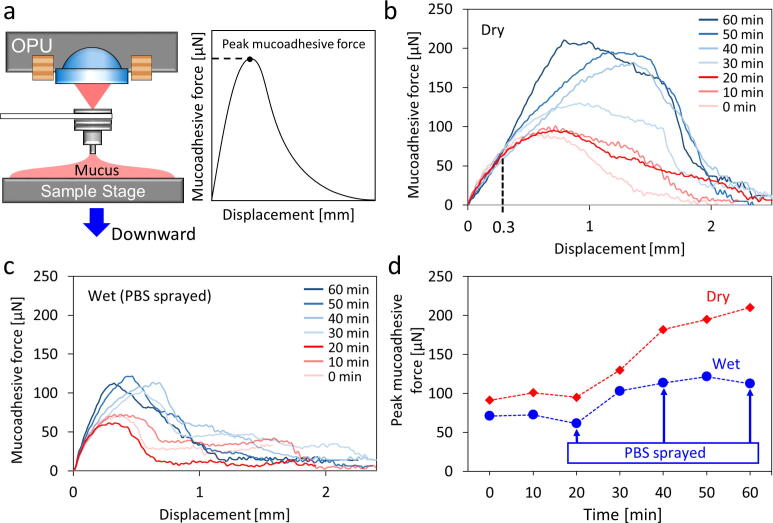
Investigation of mucoadhesive force alteration while time and moisture escapes. a) Diagram of the mucoadhesive force measurement showing that the sample stage moves downward to withdraw the mucus from the microprobe. Mucoadhesive force-to-displacement curves were measured while withdrawing a microprobe from the porcine small intestinal mucosa of **b)** dry group (no treatment) and **c)** wet group (phosphate-buffered saline (PBS) sprayed on the tissue for every 20 min), for every 10 min interval. **d)** Peak mucoadhesive force of the dry and wet groups every 10 min per hour. The mucoadhesive force of the dry group increases by 130 % after 1 h, while that of the wet group increases by 57 %.

While characterizing the mucoadhesive force of microdevices towards an intestinal mucus layer, the dehydrating effect caused the mucus layer to be more adhesive and increased the measured mucoadhesion force. The dehydrating effect usually becomes severe over time. Two experimental groups were set with a dry and wet sample to determine how time affected the adhesive behavior of the mucus layer. The mucoadhesive force of a slice of porcine small intestinal tissue was measured every 10 min using a microprobe. For the wet sample group, phosphate-buffered saline (PBS buffer) with a pH of 6.5 was constantly sprayed on the tissue to moisten the mucus layer every 20 mins for approximately 1 s. In contrast, the tissue in the dry sample group was not treated. The tissue sample was approached and withdrawn from the microprobe to directly investigate the mucoadhesive properties of the microprobe with a contact force of 80 µN, a contact time of 1 s, and an approach/withdrawal speed of 0.156 mm/s (Fig. 11**a**). The measurements were performed at room temperature of 22.5 ± 2.5 °C and relative humidity of 45 ± 5 %. The cantilever force transducer applied was 0.1 mm in thickness and 8.26 mm in length.

The mucoadhesion measurements for the dry and wet groups are shown in Fig. 11**b and c**, respectively. The results indicate that the mucoadhesive force of the groups increased with time. In the first 20 min, both groups had a relatively stable mucoadhesive force (Fig. 11**d**). The mucoadhesive force in the dry group continuously increased for 60 min. Finally, the mucoadhesive force peaked at approximately 130 % compared with the initial level. The mucoadhesion force of the wet group increased by approximately 57 % within 60 min. Furthermore, the slope of the force growing curve of the dry group (with a displacement of 0.3 mm) fluctuated at various time points (Fig. 11**b**). The wet group had the same slope (Fig. 11**c**). This preliminary study indicated that PBS treatment maintained mucus layer stability during mucoadhesive force measurements. In addition, the mucus layer behaved stable within 20 min.

### Mucoadhesive force measurement of microcontainers

Microcontainers (MCs) are polymeric cylindrical microdevices intended for oral drug delivery [1], [2]. They have a cavity for drug loading on the top side, and since only one side is open, they can provide unidirectional drug release through the GI tract to increase drug absorption efficiency. The transit mechanism has been investigated using an *ex vivo* intestinal perfusion model and animal studies [7], [21]. However, the mucoadhesion force of a single MC has not been thoroughly studied because of the limited resolution of the conventional texture analyzer.

**Fig. 12:**
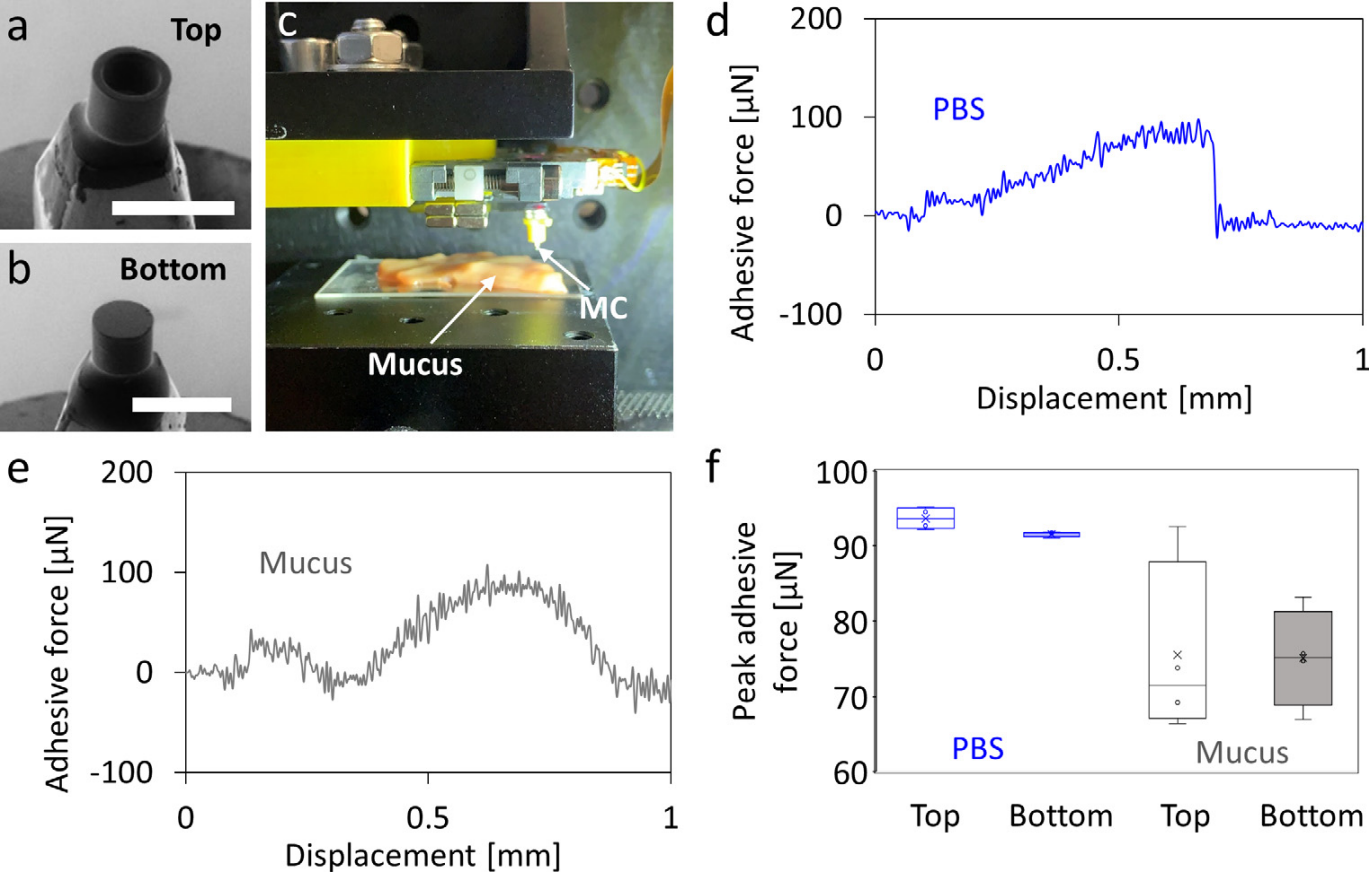
Investigation of the adhesive force of the PBS buffer and porcine small intestinal mucus to microcontainers (MCs). SEM image when mounting the MC on the microprobe with **a)** top and **b)** bottom side for measurement (scale bar represents 500 µm). **c)** Photograph when approaching and withdrawing the MC to and from the intestinal mucus with a constant speed of 0.078 mm/s and contact time of 1 s. While withdrawing the MC from the sample, the force detection module obtained the adhesive force-to-displacement curves of **d)** PBS buffer to MC and **e)** porcine small intestinal mucus to MC, respectively. **f)** The peak adhesive force of the PBS and mucus to both sides of the MC, respectively. Mean ± SD, n = 4.

In this study, the OPU force analyzer was used to measure the *ex vivo* mucoadhesive force of a single SU-8 MC using a piece of porcine small intestinal tissue. The adhesion of PBS buffer to the MC was also investigated for comparison. The MC was glued to the tip of the microprobe for contact with the sample (mucus and PBS) with the top and bottom sides, respectively (Fig. 12**a and b**). Fresh porcine small intestine tissue was stored in a freezer under −17° C. The tissue was thawed at room temperature for 20 min and sliced into a small piece of approximately 20 mm in length. The tissue was opened using scissors and placed on the sample stage with the mucosa side upward (Fig. 12**c**). In addition, a PMMA plate was placed on the sample stage to carry the PBS buffer. The pool of PBS buffer appeared to be an approximately 30-mm-diameter round and a flat surface in the center. The MC contacted the center part of PBS buffer pool for measuring the adhesive force. The parameters were set to a constant speed of 0.078 mm/s and a contact time of 1 s. Four measurements of each sample of both the top and bottom sides of the MC were implemented.

The results show that the adhesive force curve of the PBS to MC presents a right triangle (Fig. 12**d**). The adhesive force proportionally increased to an average peak force of 93.7 µN (top) and 91.6 µN (bottom) in the sample withdrawal process. As the PBS separates from the MC, the force curve returns to its original level. The mucus presented a symmetrical hill profile with an average peak force of 75.5 µN (top) and 75.1 µN (bottom) in the middle (Fig. 12**e**). Instead of sudden detachment, the mucus gradually separated from the MC. In Fig. 12**f**, the comparison indicates that the MC is more adhesive to the PBS buffer than the mucus. The measurements of the PBS buffer show relatively low variation owing to the material uniformity of the PBS buffer. In contrast, the measurements of mucoadhesive force show a high deviation. Both sides of the MC exhibit the same adhesion level, since the MC possesses a symmetrical structure on both sides. The fact that the adhesive force of the top side shows a higher degree of variation is probably because the edge of the cavity on the top side easily causes uncertain contact with mucus.

## Conclusion

• The OPU force analyzer utilized different cantilever force transducers that provided a broad range of force measurements ranging from 1.1 Newton to 0.99 nano-Newton.
• The sample-positioning module provided a 25-mm travel distance and 40-nm resolution, which had high flexibility for a wide variety of sample measurements.
• The OPU force analyzer could successfully measure the micro-Newton scale interaction force curves between a single MC and the intestinal mucus layer under different conditions.
• The simple, cost-effective, and open-source OPU force analyzer has a high potential for various force measurement applications in different fields.
• The noise of FES while measuring is approximately twice of original FES due to the environmental vibration and electrical noise. Hence, the OPU force analyzer has the potential to further extend the force range down to the pico-Newton scale by minimizing the noise of FES. The feasible ways to reduce electrical noise and interference from the environment are strengthening the mechanical rigidity, implementing an anti-vibration structure, and improving the design of electronic circuits and power source.

## Ethics statements

All ethical guidelines were complied with in this work.

## Declaration of Competing Interest

The authors declare that they have no known competing financial interests or personal relationships that could have appeared to influence the work reported in this paper.
