# Supplementary material for: Open-source force analyzer with broad sensing range based on an optical pickup unit
Source: HardwareX. 2022 Apr 20;11:e00308. doi: 10.1016/j.ohx.2022.e00308 (PMC9062582; doi:10.1016/j.ohx.2022.e00308)
Supplement: Supplementary data 1 [file mmc1.docx]

Supplementary Materials for

Open-source force analyzer with broad sensing range based on an optical pickup unit

Authors: Tien-Jen Chang*, Line Hagner Nielsen, Anja Boisen, En-Te Hwu

Affiliations:

The Danish National Research Foundation and Villum Foundation's Center for Intelligent Drug Delivery and Sensing Using Microcontainers and Nanomechanics (IDUN), Department of Health Technology, Technical University of Denmark, 2800 Kgs. Lyngby, Denmark

*corresponding author contact: tiech@dtu.dk

This PDF file includes:

Figures S1

Table S1


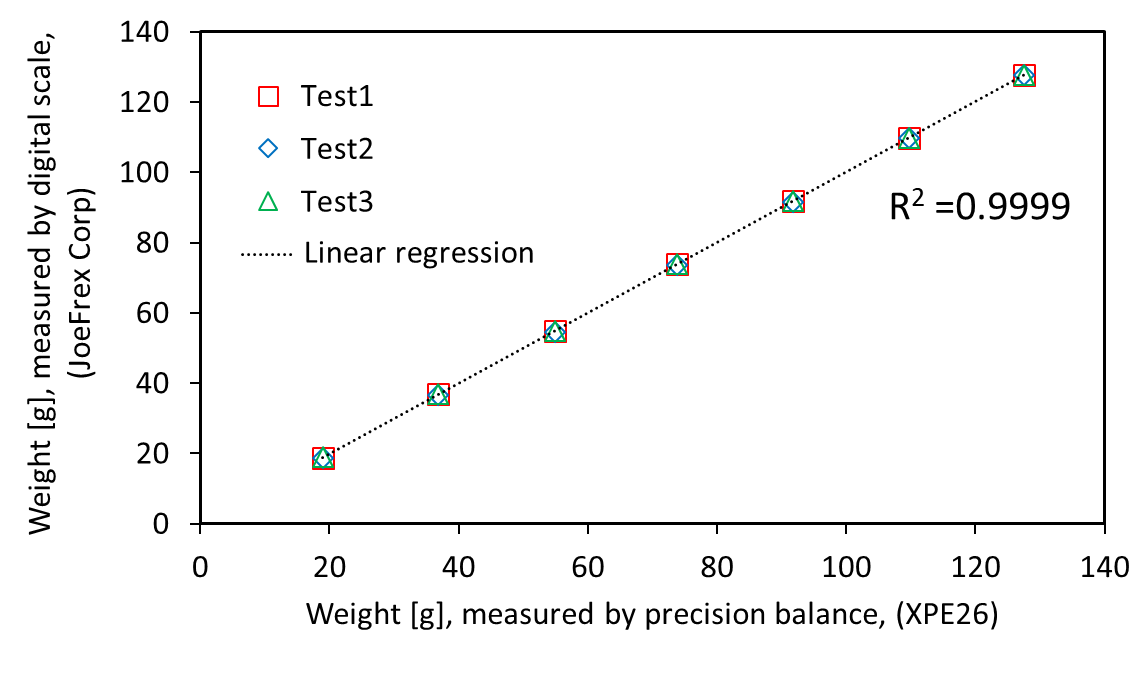


Figure S1. Weight measurement calibration of digital scale (JoeFrex Corp., Huston, United States) within the force range from 18 g to 127 g, using a precision balance (XPE26, Mettler Toledo, Greifensee, Switzerland), n=3. The data is analyzed by linear regression method, with a standard residual error of 0.08021 V and R-squared value of 0.9999.

Table S1. Repeatability and reproducibility analysis of the OPU force analyzer in the micro-Newton force range. The force analyzer characterizes the adhesion force of the bottom side of the microcontainer (diameter: 320 µm) to the distilled water, within the sample size of 5, the measurements of 3, and the trials of 3. The repeatability and reproducibility are 6.66 µN and 1.42 µN, respectively.
